# Prognostic Role of TIGIT Expression in Patients with Solid Tumors: A Meta-Analysis

**DOI:** 10.1155/2021/5440572

**Published:** 2021-11-30

**Authors:** Kunmin Xiao, Kunlin Xiao, Kexin Li, Peng Xue, Shijie Zhu

**Affiliations:** ^1^Department of Oncology, Wangjing Hospital, China Academy of Chinese Medical Sciences, Beijing, China; ^2^Graduate School, Beijing University of Chinese Medicine, Beijing, China; ^3^School of Nursing, Xinjiang Medical University, Urumqi, China

## Abstract

**Background:**

T cell immunoglobulin and ITIM domain (TIGIT) is a recently identified immunosuppressive receptor. The expression levels of TIGIT affect the prognosis of patients with solid tumors. To fully comprehend the role of TIGIT on the prognosis of patients with solid tumors, we conducted a meta-analysis.

**Methods:**

We performed an online search of PubMed, Embase, Web of Science (WOS), and MEDLINE databases for literature published till March 31, 2021. The Newcastle-Ottawa Scale (NOS) was used to evaluate the quality of the literature, and Stata 16.0 and Engauge Digitizer 4.1 software were used for data analysis.

**Results:**

Our literature search identified eight papers comprising 1426 patients with solid tumors. Increased expression of TIGIT was associated with poor prognosis. High expression of TIGIT was a risk factor for overall survival (OS) {hazard ratio (HR) = 1.66, 95% confidence interval (CI) [1.26, 2.20], *P* < 0.001} and progression-free survival (PFS) (HR = 1.44, 95% CI [1.15, 1.81], *P* = 0.01). We performed subgroup analysis to explore the source of heterogeneity, colorectal cancer (HR = 2.07, 95% CI [0.23, 18.82], *P* = 0.518), lung cancer (HR = 1.29, 95% CI [0.96, 1.72], *P* = 0.094), esophageal cancer (HR = 1.70, 95% CI [1.20, 2.40], *P* = 0.003), and other cancers (HR = 1.83, 95% CI [1.25, 2.68], *P* = 0.002). In addition to cancer type, expression location, sample size, and different statistical analysis methods are also considered the possible causes of heterogeneity between studies. Funnel plots suggested no publication bias for OS (*P* = 0.902), and Egger's test supported this conclusion (*P* = 0.537).

**Conclusion:**

TIGIT expression was associated with OS and PFS in patients with solid tumors. Patients with elevated TIGIT expression have a shorter OS and PFS, and TIGIT expression could be a novel biomarker for prognosis prediction and a valuable therapeutic target for solid tumors.

## 1. Introduction

The global burden of cancer morbidity and mortality is rapidly increasing. By 2020, there are estimated to be 19.29 million new cancer cases and 9.96 million deaths worldwide. It is expected that by 2040, the global burden of cancer will reach 28.4 million cases, an increase of 47% compared with that in 2020 [1]. In recent years, with deepening of the understanding of tumor molecular mechanisms, many tumor markers have been identified, which can be used for tumor diagnosis and prognosis judgement. Identification of new biomarkers with the potential to predict the progress and prognosis of cancer has brought new hope for cancer patients.

T cell immunoglobulin and ITIM domain (TIGIT), also known as WUCAM, Vstm3, and VSIG9, is a newly discovered coinhibitory receptor belonging to the poliovirus receptor/nectin family. TIGIT is primarily expressed in T cells and natural killer cells. Yu's group was the first to determine the unique structure of TIGIT and explore its function [2]. TIGIT is expressed in various levels in various T cell subsets. Abnormally expressed TIGIT suppresses immune cells in multiple steps of the tumor immune cycle and promotes tumor immune escape to a great extent [2–4]. Similar to the classical immune checkpoint of programmed cell death protein 1/programmed cell death ligand 1 (PD-1/PD-L1), cytotoxic T-lymphocyte-associated antigen 4 (CTLA-4)/B7.1/2 and CD226/TIGIT-CD155/CD112 are considered emerging pathways that precisely regulate T cell activation [5].

TIGIT is overexpressed in many solid tumors, including in liver cancer [6, 7], colorectal cancer (CRC) [8–10], breast cancer [11, 12], thyroid cancer [13], lung cancer [14–16], gastric cancer (GC) [17, 18], esophageal squamous cell carcinoma (ESCC) [19], and melanoma [20, 21]. Liang et al. discovered that TIGIT expression level in tumor tissue was correlated with CRC recurrence and survival [10]. TIGIT expression was considerably higher in advanced CRC than in early CRC. TIGIT expression is an independent prognostic factor for CRC and leads to a poor prognosis. However, other studies have shown that TIGIT expression is downregulated in the peripheral blood in advanced CRC, and there is no strong relationship between TIGIT expression and the overall survival (OS) rate of CRC [8, 9]. The prognosis prediction value of TIGIT for various cancers remains controversial. Therefore, we performed a meta-analysis to gain a better understanding of the impact of TIGIT on the prognosis of patients with solid cancer.

## 2. Methods

### 2.1. Search Strategies

We followed the Preferred Reporting Items for Systematic Reviews and Meta-Analyses (PRISMA) criteria. We searched PubMed, EMBASE, Web of Science (WOS), and MEDLINE databases for articles on the correlation between TIGIT expression and the prognosis of malignant tumors from the date of establishment of the database to March 31, 2021. The keywords used were “TIGIT,” “T-cell Ig and ITIM domain,” “WUCAM,” “Vstm3,” “VSIG9,” “carcinoma,” “tumor,” “neoplasia,” “neoplasm,” “cancer,” “malignancy,” “malignant neoplasm,” “prognostic,” “survival,” “prognosis,” “recurrence,” “outcome,” and “mortality.” Based on the characteristics of different databases, we conducted a comprehensive search of medical subject words (MeSH) combined with text words. The language of the restricted search was English.

### 2.2. Criteria for Inclusion and Exclusion

The inclusion criteria were as follows: (1) patients with solid tumors who underwent pathological testing to verify their diagnosis; (2) prospective or historical cohort studies; (3) immunohistochemistry (IHC) staining was used to determine TIGIT expression; (4) the cut-off value of TIGIT was reported; (5) correlation of TIGIT with survival indexes, such as OS, progression-free survival (PFS), disease-free survival (DFS), and relapse-free survival (RFS) was described; and (6) the hazard ratio (HR) and its 95% confidence interval (CI).

Exclusion criteria were as follows: (1) abstracts, reviews, case reports, letters, or nonclinical studies; (2) insufficient data for HR and 95% CI; (3) there were no studies published in English; and (4) duplicate data or analysis was identified in the studies.

### 2.3. Data Extraction and Quality Evaluation

Two independent authors (KMX and KLX) evaluated and extracted all candidate papers. In case of a dispute, the two authors consulted with a third author (KXL). The following details were extracted from the studies: first author, publication year, patient source, sample size, TIGIT positive rate, cancer type, detection method, expression location, cut-off value, statistical method, results, HR estimation method (univariate and multivariate analysis), and HR ratio. The required data were directly extracted or obtained from the survival curve using Engauge Digitizer 4.1 software to calculate the HR and 95% CI. Two independent authors (KMX and KLX) used Newcastle-Ottawa Scale (NOS) to determine the quality of the studies involved [22]. When the score was ≥6, the included literature was considered to be of high quality.

### 2.4. Statistical Methods

HR and 95% CI were pooled using Stata 16.0, to evaluate the impact of high and low TIGIT expression on the prognosis of patients with solid tumors. *I*^2^ is a quantitative statistic that reflects the percentage of interstudy variation in the overall variation [23]. According to the rule of thumb, for interpreting *I*^2^ statistics provided by the Cochrane Handbook [24], *I*^2^ ≥ 50% indicates substantial heterogeneity. The random-effects model was used when significant heterogeneity was observed; otherwise, a fixed-effects model was used. Subgroup and sensitivity analyses were used to investigate the origins of heterogeneity; Begg's and Egger's tests were used to determine publication bias. A 2-sided *P* < 0.05 was considered statistically significant.

## 3. Results

### 3.1. Study Selection

**Figure 1:**
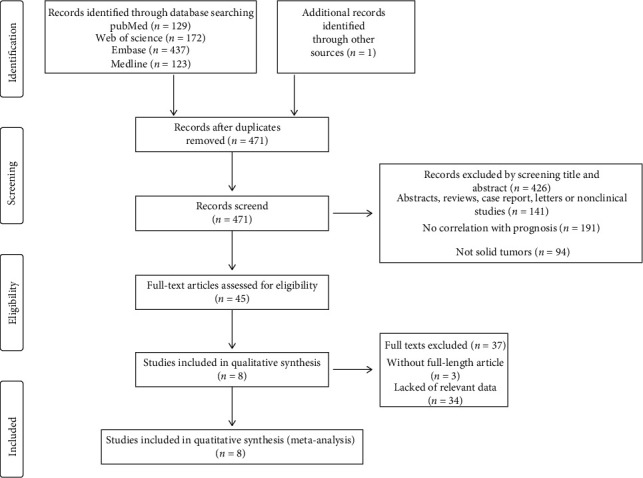
Literature screening process and results.

A total of 862 papers were initially identified. After removing duplicate literature and reading the title, abstract, and full text, according to the study's inclusion and exclusion requirements, eight papers were included [9, 10, 15–17, 19, 21, 25]. The process and results of the literature screening are presented in Figure 1.

### 3.2. Study Characteristics

Table 1 shows the basic characteristics of the studies involved in this meta-analysis. Eight articles included in this meta-analysis were published from 2018 to 2021, involving 1426 patients. All studies used IHC to detect TIGIT expression levels, but the cut-off values were not identical. The study subjects were from China (*n* = 7) and South Korea (*n* = 1). Cancer types included melanoma, lung adenocarcinoma (LUAD), GC, small cell lung cancer (SCLC), ESCC, primary small cell carcinoma of the esophagus (PSCCE), and CRC. Five studies explicitly documented reported HRs and 95% CIs, while the other three studies calculated HR and 95% CI from the survival curves. All studies evaluated the correlation between TIGIT expression and OS in patients with solid tumors [9, 10, 15–17, 19, 21, 25], and three studies evaluated the relationship between TIGIT expression and PFS in patients with solid tumors [16, 21, 25]. Zhou et al. [9] and Liang et al. [10] reported the relationship between TIGIT expression and DFS and RFS, respectively. The NOS scores of all included articles were ≥6, and the scoring details are presented in Table S1.

### 3.3. Overall Survival

**Figure 2:**
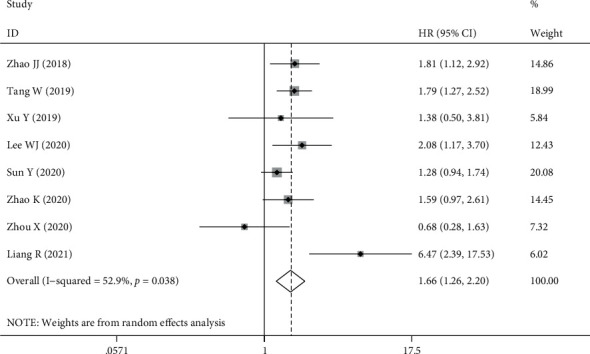
Forest plot for OS.

Eight studies provided sufficient data to investigate the connection between TIGIT expression and OS. The pooled results of the meta-analysis indicated that upregulation of TIGIT expression was correlated with worsening OS in patients with solid cancer (HR = 1.66, 95% CI [1.26, 2.20], *P* < 0.001). Heterogeneity across studies was *I*^2^ = 52.9%, *P* = 0.038; therefore, a random-effects model was used for analysis (as shown in Figure 2). Subgroup analysis was used to investigate the origin of heterogeneity. The heterogeneity of CRC (HR = 2.07, 95% CI [0.23, 18.82], *P* = 0.518) was as high as *I*^2^ = 91%, while lung cancer (HR = 1.29, 95% CI [0.96, 1.72], *P* = 0.094), esophageal cancer (HR = 1.70, 95% CI [1.20, 2.40], *P* = 0.003), and other cancers (HR = 1.83, 95% CI [1.25, 2.68], *P* = 0.002) did not show heterogeneity. The expression of TIGIT and TILs on tumor cells was significantly correlated with poor OS (tumor cells HR = 1.78, 95% CI [1.19–2.65], *P* = 0.005); tumor infiltrating lymphocytes (TILs) (HR = 1.41, 95% CI [1.09, 1.83], *P* = 0.009). Studies with sample sizes ≥100 showed a tendency to increase the risk of short OS (HR = 1.80, 95% CI [1.36, 2.39], *P* ≤ 0.001). Studies with sample sizes >100 showed the opposite trend (HR = 0.92, 95% CI [0.47, 1.78], *P* = 0.802); however, significant differences were not observed. In terms of methods for estimating HR, univariate analysis had a greater effect on prognosis than multivariate analysis (HR = 2.57, 95% CI [1.17, 5.67], *P* = 0.019 vs. HR = 1.49, 95% CI [1.24, 1.80], *P* ≤ 0.001) (Table 2).

### 3.4. Progression-Free Survival

**Figure 3:**
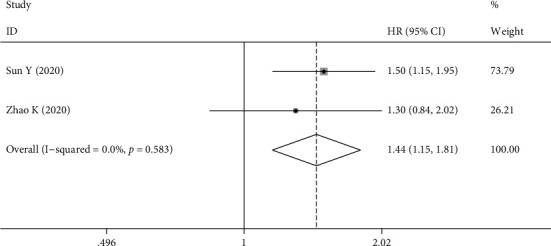
Forest plot for PFS.

Three studies involving 572 patients reported an association between TIGIT expression and PFS in patients with solid tumors, and Lee WJ analysis showed that patients with high TIGIT expression had considerably worse PFS than patients with low TIGIT expression (59.0 months vs. 32.0 months, *P* = 0.01); however, HR values and Kaplan-Meier curves were not provided; therefore, a meta-analysis could not be conducted. The fixed-effects model was used in this analysis because there was no significant heterogeneity between the studies (*I*^2^ = 0.0%, *P* = 0.583). The results showed that high expression of TIGIT was a risk factor for poor PFS (HR = 1.44, 95% CI [1.15, 1.81], *P* = 0.01). As DFS and RFS were documented in only one related article, they were not sufficient for a meta-analysis (Figure 3).

### 3.5. Sensitivity Analysis

**Figure 4:**
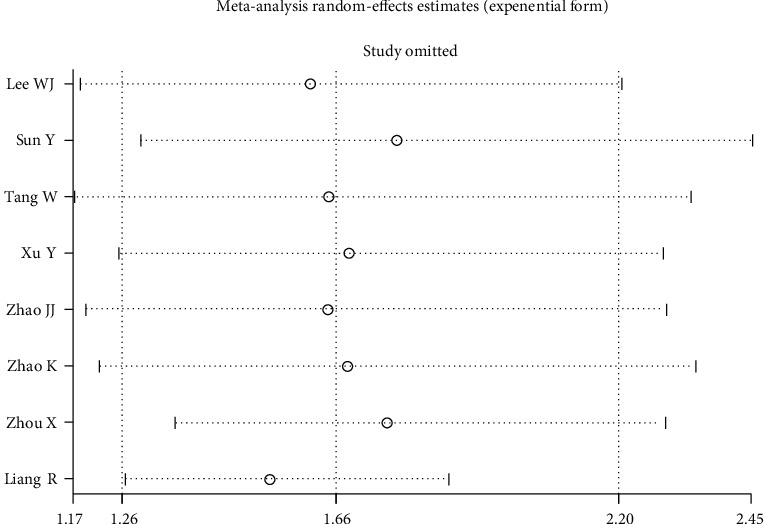
Consequence of sensitivity analysis.

Sensitivity analysis eliminated each study individually, and then, a combined analysis was conducted for the remaining studies. The results showed that the combined effect value before and after the elimination of any study had no significant change, suggesting that the results of this study were stable (as shown in Figure 4).

### 3.6. Publication Bias

**Figure 5:**
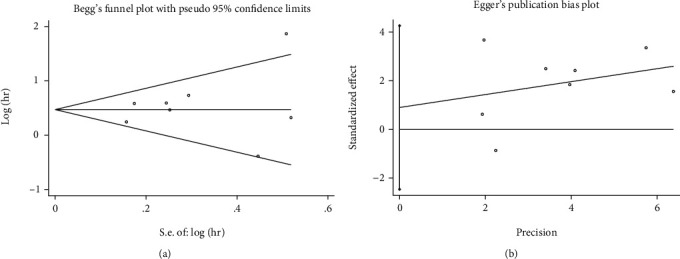
Result of publication bias. (a) Begg's test; (b) Egger's test.

Begg's and Egger's methods were used to assess the publication bias. The funnel plot revealed no major asymmetry (*P* = 0.902; Figure 5(a)). Furthermore, Egger's test supported this conclusion (*P* = 0.537; Figure 5(b)). Therefore, our meta-analysis did not reveal any publication bias.

## 4. Discussion

TIGIT is a fairly new immunosuppressive receptor, discovered 11 years ago. In recent years, TIGIT expression has been shown to have prognostic significance in patients with solid tumors in a variety of trials, but its role has been inconsistent and unclear. Therefore, we reviewed published studies and performed a meta-analysis. Our present meta-analysis may be the only study to date evaluating the association between TIGIT expression and OS in patients with solid tumors.

Our meta-analysis included 1430 patients with solid tumors from eight studies. The results showed that increased expression of TIGIT was associated with a poor prognosis. High expression of TIGIT was a risk factor for OS (HR = 1.66, 95% CI [1.26, 2.20], *P* < 0.001) and PFS (HR = 1.44, 95% CI [1.15, 1.81], *P* = 0.01). In addition, we found that the cancer type, expression location, sample sizes, and different statistical analysis methods are possible reasons for the heterogeneity between studies. Our results emphasize the prognostic value of TIGIT expression in patients with solid tumors.

The immune checkpoint is one of the main causes of immune tolerance. Immunotherapy targeting the classical immune checkpoints of PD-1/PD-L1 and CTLA-4/B7.1/2 has brought hope to patients with tumors. However, in actual clinical applications, only a part of the dominant population has an immune response, and PD-1/PD-L1 immune checkpoint inhibitors are prone to drug resistance and severe adverse effects [26, 27]. Therefore, there is an urgent need to identify new immune checkpoints to compensate for low response rates, drug resistance, and severe adverse reactions, such as lymphocyte activation gene 3 (LAG-3) [28], T cell immunoglobulin-3 (TIM-3) [29], and TIGIT, which can negatively regulate T cell activation and function and induce T cell exhaustion; however, they each have unique signaling pathways and regulatory functions, so their clinical applications are different [30]. Compared with those on LAG-3 and TIM-3, studies on TIGIT expression provided more encouraging results [31]. TIGIT has a higher positive rate in TILs than does PD-1 [19], and TIGIT inhibitors can transduce CD155-mediated signals to CD226 activation, thus improving immunotherapy, which is advantageous [20]. In CITYSCAPE, a phase II clinical trial, 135 patients with PD-L1 positive non-small-cell lung cancer were randomized to receive the TIGIT inhibitor tiragolumab in combination with a PD-L1 inhibitor or PD-L1 inhibitor alone. The results showed that addition of tiragolumab significantly improved patient outcomes, with the objective response rate increasing from 21% to 37% and the median PFS increasing from 3.9 months to 5.6 months without an increase in adverse events. In particular, the objective response rate of patients with high PD-L1 expression (>50%) increased from 24% to 66% after the addition of tiragolumab [32]. In addition to the combination of PD-1/PD-L1 inhibitors, the combination of TIGIT antibody and other immune checkpoint inhibitors can also produce synergistic effects and improve the efficacy of immunotherapy [33].

TIGIT affects the prognosis of cancer patients by inhibiting the function of immune cells through a variety of mechanisms [34]. These are as follows: (1) TIGIT binds to CD155 and causes T cells to send a direct inhibition signal, inducing immune tolerance [35]; (2) TIGIT stimulates the immune response indirectly by activating CD155 on DCs, increasing IL-10 secretion while decreasing IL-12 secretion [2]; (3) TIGIT not only competes with the CD226 ligand but also binds directly to CD226 and disrupts its homodimerization, thereby disrupting its costimulatory effect and preventing CD226-mediated T cell activation [4]; (4) TIGIT signaling in regulatory T cells (Tregs) affects the secretion of cytokines and suppresses proinflammatory Th1 and Th17 T cell responses [36], which enhances the immunosuppressive function and stability of Tregs.

We showed an association between TIGIT expression and the prognosis of patients with solid tumors, but the prognostic effects of TIGIT on different tumors were inconsistent, which may be due to different characteristics and different expression sites of different tumors. Significant heterogeneity was observed in the two CRC studies, which could be explained by study's methodological design and confounders of clinical covariates. PFS, DFS, and RFS can all predict and reflect clinical benefits, but there are few relevant studies reported at present, and it is impossible to measure the combined HR. Many studies are needed to evaluate the prognostic value of TIGIT.

Although we tried our best to conduct a comprehensive analysis, our meta-analysis has certain limitations. Despite the use of subgroup and sensitivity analyses, the origin of the heterogeneity could not be completely traced. Second, all the studies were retrospective, with all subjects being Asian, which does not represent the whole population. Third, the scale of the included studies was small. Some studies include only one kind of cancer, and studies with a larger sample size are required to fully understand the connection between TIGIT and the survival index. Third, although IHC was used in all the studies, the antibodies used were no identical, and the thresholds were not consistent. We should further explore the establishment of a unified threshold. Fourth, the number of studies included in this article was very limited. When the number of included articles is less than 10, the efficiency of Begg's test and Egger's test detection tend to reduce, and as this study included English language articles, publication bias cannot be ruled out. We hope that this meta-analysis not only represents the end point of the study but also begins to pay attention to the value of TIGIT in solid tumors and to look forward to more high-quality studies.

## 5. Conclusion

Taken together, our findings indicate a significantly increased risk of OS and PFS associated with elevated TIGIT expression. TIGIT appears to be a promising therapeutic target for solid tumors as well as a prognostic predictor, which deserves the attention of researchers and clinicians. Due to the limitations of the number and quality of the included literature, our results need to be interpreted carefully. Further studies are necessary to evaluate the molecular mechanism of TIGIT in patients with solid tumors.

## Figures and Tables

**Table 1:** Basic characteristics of included studies.

Author	Year	Patient source	Sample size	TIGIT +	Cancer type	Method	Expression location	Cut-off value	Outcome	M/U	HR ratio	NOS
Zhao JJ	2018	Chinese	154	76 (49.4%)	ESCC	IHC	TIL	Median level	OS	M	Reported	8
Tang W	2019	Chinese	441	343 (77.8%)	GC	IHC	Tumor cell	>5% positivity cell	OS	M	Reported	8
Xu Y	2019	Chinese	60	21 (35%)	SCLC	IHC	Tumor cell	Median level	OS	U	Survival curve	6
Lee WJ	2020	Korea	124	52 (41.9%)	Melanoma	IHC	Tumor cell	≥20% positivity cell	OS/PFS	U	Survival curve	7
Sun Y	2020	Chinese	334	204 (61.1%)	LUAD	IHC	TIL	≥5% positivity cell	OS/PFS	M	Reported/survival curve	7
Zhao K	2020	Chinese	114	74 (64.9%)	PSCCE	IHC	Tumor cell	≥5% positivity cell	OS/PFS	M	Reported/survival curve	8
Zhou X	2020	Chinese	60	21 (35%)	CRC	IHC	Tumor cell	Score ≥ 1	OS/DFS	M	Reported	8
Liang R	2021	Chinese	139	40 (28.8%)	CRC	IHC	Tumor cell	Median level	OS/RFS	U	Survival curve	6

OS: overall survival; PFS: progress-free survival; DFS: disease-free survival; RFS: recurrence-free survival; U: univariate; M: multivariate; TIL: tumor infiltrating lymphocyte; IHC: immunohistochemistry; LUAD: lung adenocarcinoma; GC: gastric cancer; SCLC: small cell lung cancer; ESCC: esophageal squamous cell carcinoma; PSCCE: primary small cell carcinoma of the esophagus; CRC: colorectal cancer.

**Table 2:** Subgroup analysis results.

		Random-effects model		Fixed-effects model		Heterogeneity	
Analysis	*N*	HR (95% CI)	*P*	HR (95% CI)	*P*	*I* ^2^	pH
*Cancer type*							
Colorectal cancer	2	2.07 (0.23, 18.82)	0.518	1.81 (0.94, 3.50)	0.076	91.0%	0.001
Lung cancer	2	1.29 (0.96, 1.72)	0.094	1.29 (0.96, 1.72)	0.094	0.0%	0.886
Esophagus cancer	2	1.70 (1.20, 2.40)	0.030	1.70 (1.20, 2.40)	0.003	0.0%	0.715
Others	2	1.86 (1.39, 2.50)	0.001	1.86 (1.39, 2.50)	0.001	0.0%	0.662
*Expression position*							
Tumor cells	6	1.78 (1.19, 2.65)	0.005	1.77 (1.40, 2.22)	0.001	57.7%	0.037
TILs	2	1.44 (1.04, 2.00)	0.027	1.41 (1.09, 1.83)	0.009	30.2%	0.231
*Sample size*							
<100	2	0.92 (0.46, 1.83)	0.817	0.92 (0.47, 1.78)	0.802	6.5%	0.301
≥100	6	1.80 (1.36, 2.39)	0.001	1.67 (1.40, 1.99)	0.001	54.1%	0.054
*Method to estimate HR*							
Multivariate analysis	5	1.49 (1.17, 1.88)	0.001	1.49 (1.24, 1.80)	0.001	31.9%	0.209
Univariate analysis	3	2.57 (1.17, 5.67)	0.019	2.42 (1.54, 3.78)	0.001	61.4%	0.075

## Data Availability

No new data generated.

## Supplementary Materials

Supplementary Materials Table S1: Newcastle-Ottawa Scale for assessing the quality of studies in meta-analysis.
