# Supplementary material for: Prognostic Role of TIGIT Expression in Patients with Solid Tumors: A Meta-Analysis
Source: J Immunol Res. 2021 Nov 30;2021:5440572. doi: 10.1155/2021/5440572 (PMC8651431; doi:10.1155/2021/5440572)
Supplement: Supplementary Materials — Table S1: Newcastle-Ottawa Scale for assessing the quality of studies in meta-analysis. [file 5440572.f1.docx]

**Table S1. Newcastle-Ottawa Scale for assessing the quality of studies in meta-analysis**

| **Study** | **Selection** | | | | **Comparability** | **Exposure** | | | **Scores** |
| --- | --- | --- | --- | --- | --- | --- | --- | --- | --- |
|  | Representativeness  of the exposed cohort | Selection of  the nonexposed cohort | Ascertainment of exposure | Demonstration that outcome of interest was not present at start of study | Comparability of cohorts on the basis of the design or analysis | Assessment of outcome | Was follow-up long enough for outcomes to occur | Adequacy of follow up of cohorts |  |
| Zhao JJ *el al*, 2018 | ★ | ★ | ★ | - | ★★ | ★ | ★ | ★ | 8 |
| Tang W *el al*, 2019 | ★ | ★ | ★ | - | ★★ | ★ | ★ | ★ | 8 |
| Xu Y *el al*, 2019 | ★ | ★ | ★ | - | ★★ | - | - | ★ | 6 |
| Lee WJ *et al*,2020 | ★ | ★ | ★ | - | ★★ | - | ★ | ★ | 7 |
| Sun Y *el al*, 2020 | ★ | ★ | ★ | - | ★★ | ★ | - | ★ | 7 |
| Zhao K *el al*, 2020 | ★ | ★ | ★ | - | ★★ | ★ | ★ | ★ | 8 |
| Zhou X *el al*, 2020 | ★ | ★ | ★ | - | ★★ | ★ | ★ | ★ | 8 |
| Liang R *el al*, 2021 | ★ | ★ | ★ | - | ★★ | - | - | ★ | 6 |

Identify ‘high’ quality choices with a ‘star’. A maximum of one ‘star’ for each item within the ‘Selection’ and ‘Exposure’ categories; maximum of two ‘stars’ for ‘Comparability’. When the ‘stars’ add up to ≥ 6 for a single literature, the included literature is considered to be of high quality.
